# Vaccination-first immune priming shapes a sustained mRNA vaccine-induced IgG4 class switch that associates with SARS-CoV-2 breakthrough infection risk

**DOI:** 10.1038/s41598-026-64190-8

**Published:** 2026-08-12

**Authors:** Tamás Pongrácz, Ulrika Marking, Oscar Bladh, Katherina Aguilera, Matilda Berkell, Sebastian Havervall, Nina Greilert-Norin, Jan Nouta, Steinar Gijze, Anna Wasynczuk, Mikael Åberg, Gestur Vidarsson, Youjia Zhong, David Falck, Charlotte Thålin

**Affiliations:** 1 Department of Clinical Sciences, Danderyd Hospital, Karolinska Institutet, Stockholm, Sweden; 2 National Bioinformatics Infrastructure Sweden, Stockholm, Sweden; 3 Center for Proteomics and Metabolomics, Leiden University Medical Center, Leiden, The Netherlands; 4 Department of Medical Sciences, Clinical Chemistry, Uppsala University, Uppsala, Sweden; 5 Science for Life Laboratory, Uppsala University, Uppsala, Sweden; 6 Immunoglobulin Research Laboratory, Sanquin Research, Amsterdam, The Netherlands; 7 Department of Biomolecular Mass Spectrometry and Proteomics, Utrecht Institute for Pharmaceutical Sciences and Bijvoet Center for Biomolecular Research, Utrecht University, Utrecht, The Netherlands; 8 Department of Paediatrics, Yong Loo Lin School of Medicine, National University of Singapore, Singapore, Singapore; 9 Khoo Teck Puat-National University Children’s Medical Institute, National University Health System (NUHS), Singapore, Singapore

**Keywords:** mRNA vaccination, IgG4, Class switch, Breakthrough infection risk, IgG glycosylation, Diseases, Immunology

## Abstract

Repeated administration of mRNA vaccines against SARS-CoV-2 has been associated with qualitative changes in antibody responses, including the emergence of non-inflammatory IgG4 subclasses and changes in Fc glycosylation. While neutralizing antibody titers remain key correlates of protection, potential functional implications of these structural antibody features warrant further investigation across age groups and multiple mRNA vaccine boosters. We characterized spike-specific IgG responses—including subclasses and Fc glycosylation patterns—using a liquid chromatography—mass spectrometry-based approach across six mRNA vaccine doses in a Swedish healthcare worker cohort (n = 104) and across three mRNA doses in a Singaporean pediatric cohort (n = 18). Repeated mRNA vaccination induced an IgG4 class switch which was sustained across at least six doses. This response associated with an increased risk of breakthrough infection (HR = 1.83, p = 0.028) in infection-naïve individuals (n = 41) lacking mucosal IgA responses that would potentially confound the interpretation of systemic IgG4‑related infection outcomes. Moreover, the IgG4 class switch was preceded with high early IgG1 Fc fucosylation signatures. Notably, these IgG structural changes were observed primarily in individuals who were naïve to SARS-CoV-2 at the time of first vaccination. Similar IgG structural features were observed across three mRNA doses in the small infection-naive pediatric cohort. Our results illustrate an IgG4-dominated immunological signature in both adults and children and highlight that immune priming by prior infection may shape subsequent mRNA vaccine-induced antibody responses. While these findings do not call into question the efficacy or safety of the widely adapted mRNA vaccine platform, they may have implications that merit further investigation.

**Supplementary Information:**

The online version contains supplementary material available at 10.1038/s41598-026-64190-8.

## Introduction

A range of vaccines targeting the spike (S) protein of severe acute respiratory syndrome coronavirus 2 (SARS-CoV-2) were rapidly developed and deployed during the coronavirus disease 2019 (COVID-19) pandemic^1–4^, providing robust protection against severe disease and hospitalization^5,6^. Vaccine-induced spike-specific humoral immunity is predominantly mediated by early IgM responses that undergo class switch to give rise to IgG3 and then IgG1 subclasses, all of which bind or neutralize the virus via the antigen-binding (Fab) regions and facilitate viral clearance through Fc receptor (FcR) engagement and complement activation^7^. High neutralizing anti-spike IgG titers are highly regarded as key correlates of protection^8^, yet structural antibody features such as IgG subclass and Fc glycosylation are emerging as additional determinants of antibody function although their translation to protection remains poorly understood^9–13^.

IgG4 class switching is a well-described immunological adaptation to chronic or repeated antigen exposure linked to immune tolerance. IgG4 exhibits reduced engagement with FcγRs and complement components^10^, resulting in attenuated Fc-mediated effector functions such as antibody-dependent cellular cytotoxicity (ADCC), phagocytosis (ADCP), and complement activation^14^. Furthermore, circulating IgG4 undergo Fab-arm exchange, rendering them bispecific and limiting their capacity to form immune complexes. Repeated mRNA vaccination, unlike adenoviral vector^15–17^ or protein subunit^18,19^ platforms has been reported to promote the emergence of anti-spike IgG4^14,15,20–28^. Recent evidence has further linked elevated non-cytophilic/cytophilic IgG ratios to an increased risk of breakthrough infections^29^ and to a more severe clinical phenotype^30^, illustrating the potential impairment of Fc-mediated viral clearance following multiple mRNA doses if IgG4 becomes dominant over time^31^.

Beyond subclass, IgG functionality is further defined by Fc glycosylation^9^. For example, afucosylated IgG enhances binding to FcγRIIIa (V158)—expressed on natural killer cells, macrophages, and neutrophils—thereby amplifying ADCC^9,32^. It has been demonstrated that Fc glycosylation patterns of spike-specific IgG antibodies vary depending on prior immune history and the second dose of the primary vaccination regimen^33,34^; however, the long-term evolution of Fc glycosylation following multiple mRNA booster doses remains poorly characterized.

Importantly, a coordinated shift in IgG subclass distribution and Fc glycosylation may jointly influence the qualitative nature of the humoral response following repeated mRNA vaccine doses. To address this, we investigated IgG subclass and Fc glycosylation dynamics within the same individuals across six mRNA vaccine doses in a Swedish healthcare worker cohort (n = 104) and across three mRNA vaccine doses in a Singaporean pediatric cohort (n = 18), both well characterized with respect to infection histories prior to and between mRNA vaccinations. In addition, continuous infection monitoring allowed to examine whether distinct IgG structural features, including subclass composition and Fc glycosylation profiles, were associated with the risk of subsequent SARS-CoV-2 breakthrough infection risk, thereby linking antibody structure to clinical outcomes following repeated mRNA vaccination.

## Results

To explore IgG4 class switch and Fc glycosylation changes across repeated mRNA vaccinations and age groups, we analyzed 831 longitudinal serum samples from 104 immunocompetent adults enrolled in the ongoing COMMUNITY healthcare worker (HCW) cohort, Stockholm, Sweden, and 67 longitudinal serum samples from 18 immunocompetent children enrolled in the Singaporean MARVELS pediatric vaccination cohort **(**Table 1**)**. COMMUNITY HCWs received between five and six SARS-CoV-2 mRNA vaccine doses between January 2021 and December 2023 and MARVELS children received two to three SARS-CoV-2 mRNA vaccine doses between December 2021 and December 2022.

**Table 1:** Study cohort characteristics.

	COMMUNITY (adults)	MARVELS (children)
Number of participants	104	18
Number of samples	831	67
Age at enrolment (Median (Q1–Q3) [Min–Max])	61.0 (52.8–64.3) [30.0–69.0]	8.50 (7.25–10.8) [5.00–11.0]
Sex (n, %)		
Female	93 (89.4%)	7 (38.9%)
Male	11 (10.6%)	11 (61.1%)
Infection prior to first vaccine (n, %)		
No (vaccination-first)	65 (62.5%)	18 (100%)
Yes (infection-first)	39 (37.5%)	0 (0%)
Vaccine doses (n, %)		
2	0 (0%)	5 (27.8%)
3	0 (0%)	13 (72.2%)
5	79 (76%)	0 (0%)
6	25 (24%)	0 (0%)
Infections until last follow-up (n, %)		
0	34 (32.7%)	9 (50%)
1	46 (44.2%)	9 (50%)
2	24 (23.1%)	0 (0%)
Days between vaccine doses—median (Q1–Q3) [Min–Max]		
First to second	21.0 (21.0–41.0) [21.0–159]	22.5 (21.3–25.5) [21.0–56.0]
Second to third	259 (194–303) [95.0–336]	282 (272–289) [237–331]
Third to fourth	244 (174–282) [129–392]	N/A
Fourth to fifth	352 (167–421) [112–538]	N/A
Fifth to sixth	383 (329–406) [139–447]	N/A
Vaccine 1 type		
BNT162b	104 (100%)	18 (100%)
Vaccine 2 type		
BNT162b	103 (99.0%)	18 (100%)
mRNA-1273	1 (1.0%)	0 (0%)
Vaccine 3 type		
BNT162b	58 (55.8%)	13 (100%)
mRNA-1273	46 (44.2%)	0 (0%)
Vaccine 4 type		N/A
BNT162b BA.1	5 (4.8%)	
BNT162b BA.4–5	15 (14.4%)	
BNT162b	43 (41.3%)	
mRNA-1273 BA.1	6 (5.8%)	
mRNA-1273	35 (33.7%)	
Vaccine 5 type		N/A
BNT162b BA.1	8 (7.7%)	
BNT162b BA.4–5	25 (24.0%)	
BNT162b	4 (3.8%)	
BNT162b XBB.1.5	57 (54.8%)	
mRNA-1273 BA.1	3 (2.9%)	
mRNA-1273	7 (6.7%)	
Vaccine 6 type		N/A
BNT162b BA.4–5	2 (8%)	
BNT162b XBB.1.5	23 (92%)	

### Progressive and saturable IgG4 class switch following repeated mRNA vaccination in adults

We first characterized longitudinal anti-spike trimer IgG subclass levels across six mRNA vaccine doses in the COMMUNITY HCW cohort using a liquid chromatography-mass spectrometry (LC–MS)-based quantification approach which is comparable to established enzyme-linked immunosorbent assays, but cannot distinguish between IgG2 and IgG3^35^. Participants were stratified based on SARS-CoV-2 immune history prior to the first vaccine dose. Two groups were defined: no confirmed infection prior to first vaccine dose (vaccination-first; n = 65) and confirmed infection prior to first vaccine dose (infection-first; n = 39), respectively.

**Fig. 1:**
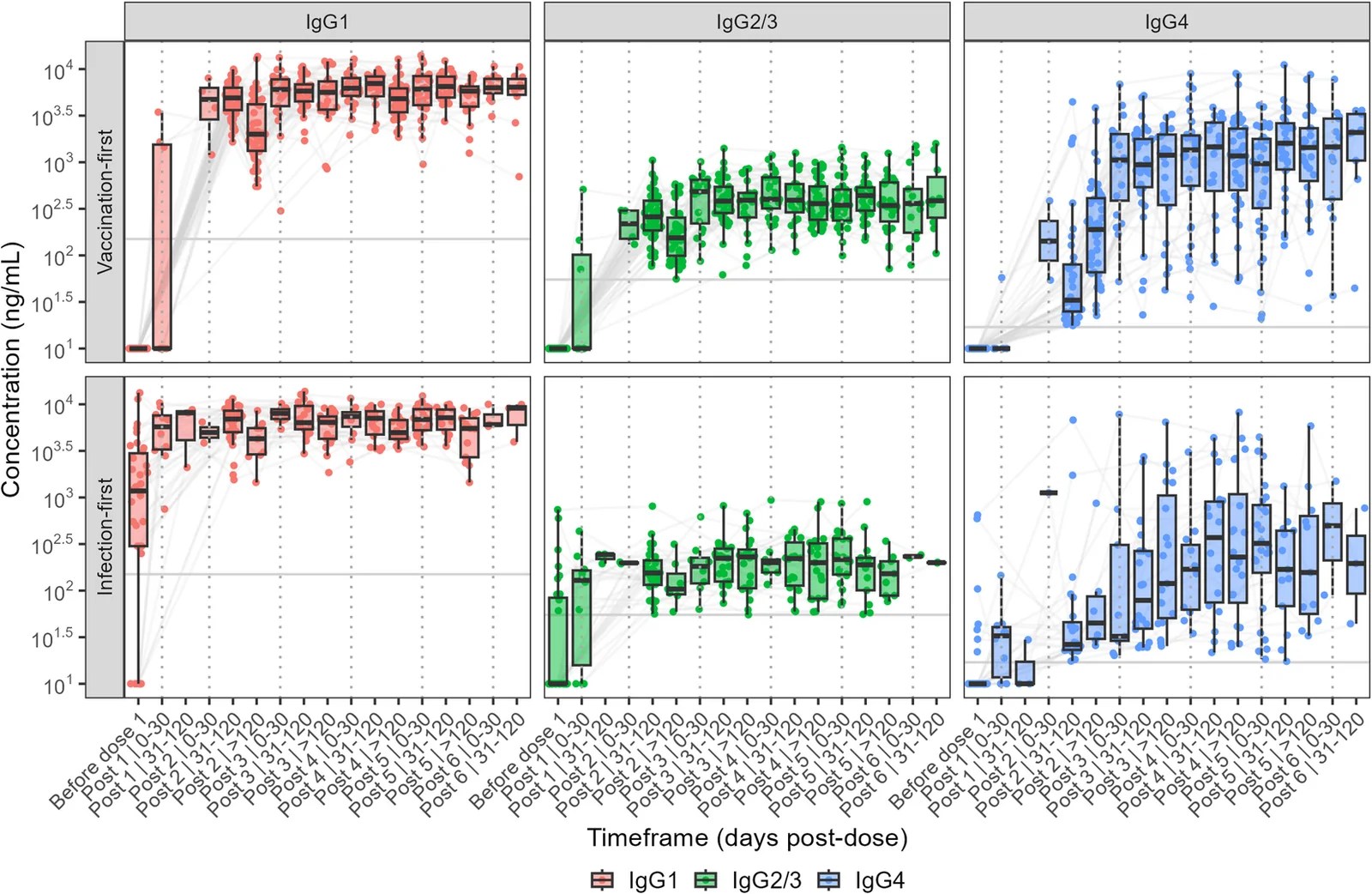
Spike-specific IgG subclass levels before the first and across 6 doses of mRNA vaccination in adults without (vaccination-first) and with (infection-first) a documented SARS-CoV-2 infection prior to vaccination. Absolute IgG subclass concentrations measured by LC–MS following each mRNA vaccine dose, stratified by post-vaccination sampling timeframes as: before vaccine dose 1; early (0–30 days); intermediate (31–120 days); and late (>120 days). Lower limit of quantification (LLoQ) indicated with horizontal lines for IgG1: 150 ng/mL; IgG2/3: 55 ng/mL; IgG4: 17 ng/mL. All IgG subclasses were significantly higher at all post-vaccination timepoints relative to timepoints before vaccine 1, following correction for multiple comparisons using the Benjamini–Hochberg procedure (false discovery rate = 5%) of Wilcoxon signed-rank test derived p-values. A timepoint corresponding to Post 6 | >120 is not shown due to low number of samples (n = 1). Dotted vertical lines denote the first timepoint after each vaccine dose.

As expected, the primary vaccination series elicited robust IgG1 and IgG2/3 responses in both vaccination-first and infection-first individuals, which were maintained over the long-term follow-up (all timepoints showed significantly higher IgG1 and IgG2/3 levels compared to the reference timepoint “Before dose 1”; p < 0.05) **(**Fig. 1**)**. An IgG4 response was observed following the second dose (Post 2 | 30–120 days; all timepoints with detectable IgG4 significantly differed from the reference timepoint “Before dose 1”; p < 0.05). Some infection-first individuals had detectable IgG4 already before the first vaccine dose and these levels increased thereafter (p < 0.05). Notably, some participants’ IgG4 concentrations approached those of IgG1 (1–10 µg/mL) **(**Fig. 1**)**, indicating a profound shift in subclass distribution over time.

### Immune history prior to the first vaccine dose influences the proportional magnitude of a saturable IgG4 response

**Fig. 2:**
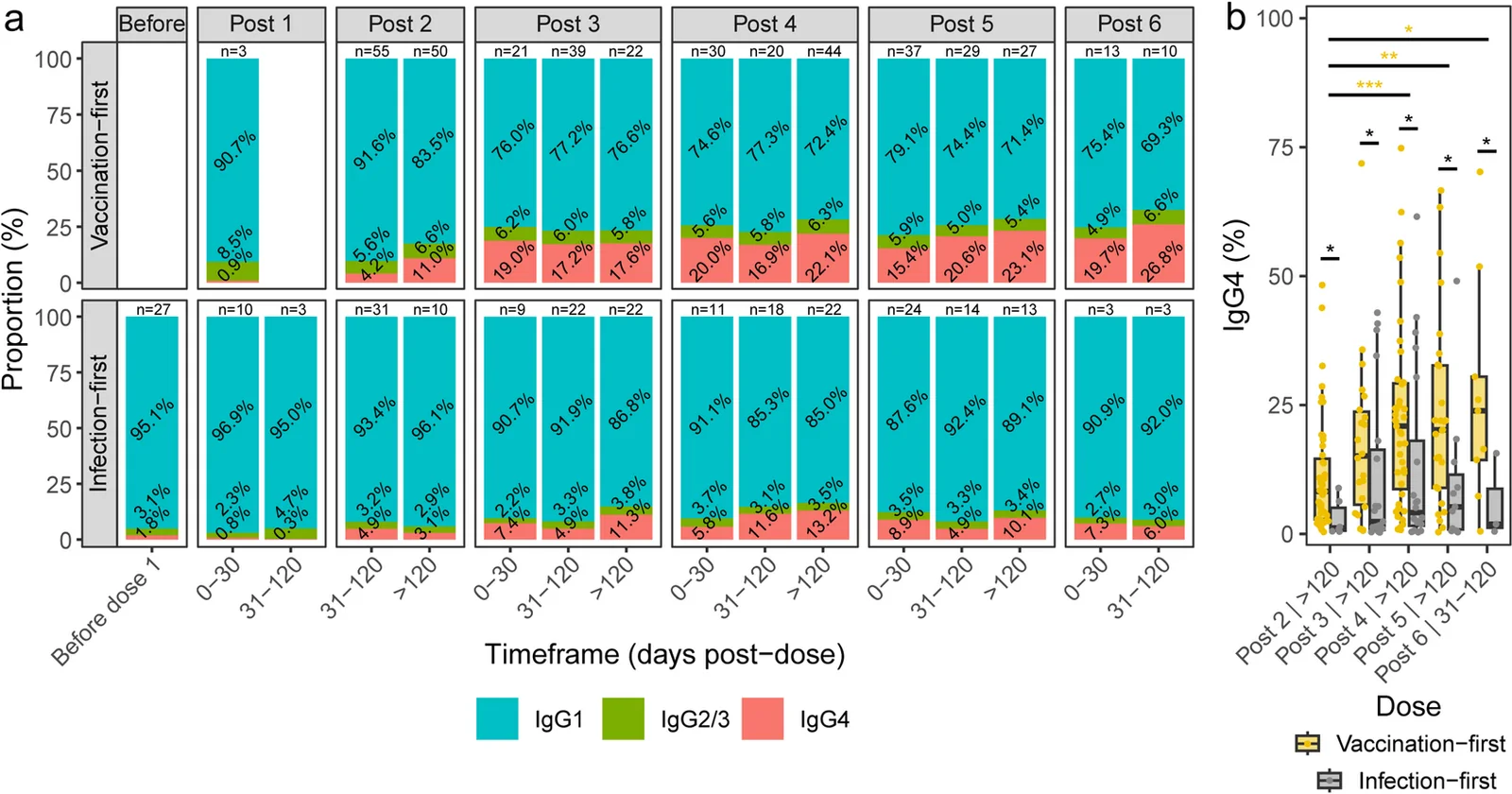
Spike-specific IgG subclass proportions before the first and across 6 doses of mRNA vaccination in adults without (vaccination-first) and with (infection-first) a documented SARS-CoV-2 infection prior to vaccination. **(a)** Proportion of IgG subclasses per post-vaccination timeframe. **(b)** Comparison of IgG4 subclass proportions between and within vaccination-first (yellow) and infection-first (grey) adults at the latest available post-dose timepoints across 6 vaccine doses. Between-group comparisons at each timepoint were performed using one-tailed Wilcoxon signed-rank tests. Within-group comparisons across timeframes were assessed using Kruskal–Wallis tests followed by post-hoc Dunn’s tests (asterisks are color-coded by group according to legend). Adjusted p-values from Kruskal–Wallis tests: vaccination-first = 0.0045; infection-first = 0.8. Significance levels: * p < 0.05, ** p < 0.01, *** p < 0.001. Only significant associations are shown. Comprehensive statistical summary in Table S1.

To better track the evolution of the IgG4 response, we calculated the proportion of each IgG subclass per participant and expressed corresponding means following each vaccine dose and post-vaccination timeframe. Stratified analysis revealed that the IgG4 class switch was dominant in vaccination-first participants, with IgG4 proportions reaching ∼11% 120 days after the second vaccine dose, and peaked at 26.8% 31–120 days after the sixth dose (Fig. 2a). The spike-specific IgG2/3 response varied between 0.9 and 6.6%.

In contrast, the IgG4 class switch was less pronounced in infection-first individuals: the highest mean IgG4 proportion was 13.2% (>120 days after the fourth dose), and hardly exceeded 10% after subsequent doses. IgG2/3 proportions ranged between 0.3 and 3.8% (Fig. 2a).

To further explore these dynamics, we compared the two groups side-by-side at the available late timepoints. As expected, accumulation of IgG4 with increasing number of vaccinations was observed in vaccination-first individuals, whereas this trend was less pronounced and did not reach statistical significance within the infection-first group (Fig. 2b). Moreover, IgG4 proportions in vaccination-first individuals consistently and substantially exceeded those in the infection-first group (Fig. 2b), but the proportional magnitude saturated after the third dose (Fig. 2b). Of note, several participants developed IgG4 dominant responses exceeding 25% reaching up to 75% (Fig. 2b). We hypothesized that shorter dose intervals may boost IgG4 proportions, but exploratory correlation analyses did not confirm this assumption (Fig. S1). We also assessed whether breakthrough infections between the vaccine doses would dampen the build-up of IgG4, and breakthrough infections were associated with a slight but statistically significant reduction on long-term IgG4 proportions in vaccination-first individuals (Fig. S2).

### High IgG4 proportions associate with subsequent breakthrough infection risk in infection-naïve individuals

**Fig. 3:**
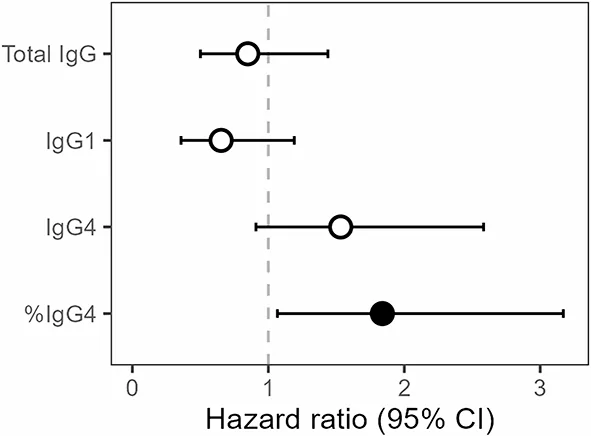
Association of total IgG, IgG1 and IgG4 levels and IgG4 proportions with the risk of breakthrough infections over a 200-day follow-up period after the third vaccine dose in vaccination-first participants who had remained infection-naïve at the time of sampling after the third vaccine dose. Hazard ratios were estimated using multivariate Cox proportional hazards models. Age and days since the third vaccine dose were included as covariates into all models. All individuals in this study were healthy females, received wild-type targeting mRNA vaccines and were exposed to similar occupational risk factors during the same calendar period and variant wave during the study period (from 2021–12-26 to 2022–04-25). The hazard ratio represents the change in the relative hazard associated with one SD increase of (total) Ig levels or IgG4 proportions.

We next proceeded to explore potential associations between high IgG4 proportions and the risk of subsequent breakthrough infection. We constricted this analysis to a limited set of 41 triple-vaccinated vaccination-first individuals since this is where the IgG4 class switch was dominant. As only infection-naïve individuals were included in the analysis, (Fig. S3) this approach also provided the basis to examine individuals exclusively relying on systemic immunity, since we and others have shown a clear link between infection-induced upper airway antibodies and protection against infection^36–41^. We found IgG4 proportions, but not total IgG, IgG1 and IgG4 levels, associated with SARS-CoV-2 breakthrough infection risk (Hazard ratio = 1.83 [95% CI = 1.07–3.17], p = 0.028) (Fig. 3).

### Anti-spike IgG1 fucosylation reflects immune history prior to the first vaccine dose, displays long-term stability, and correlates with the IgG4 class switch magnitude

We then proceeded to investigate IgG Fc glycosylation dynamics focusing on core fucosylation and galactosylation which are the two Fc glycosylation features with established roles in modulating Fc-mediated effector functions.

**Fig. 4:**
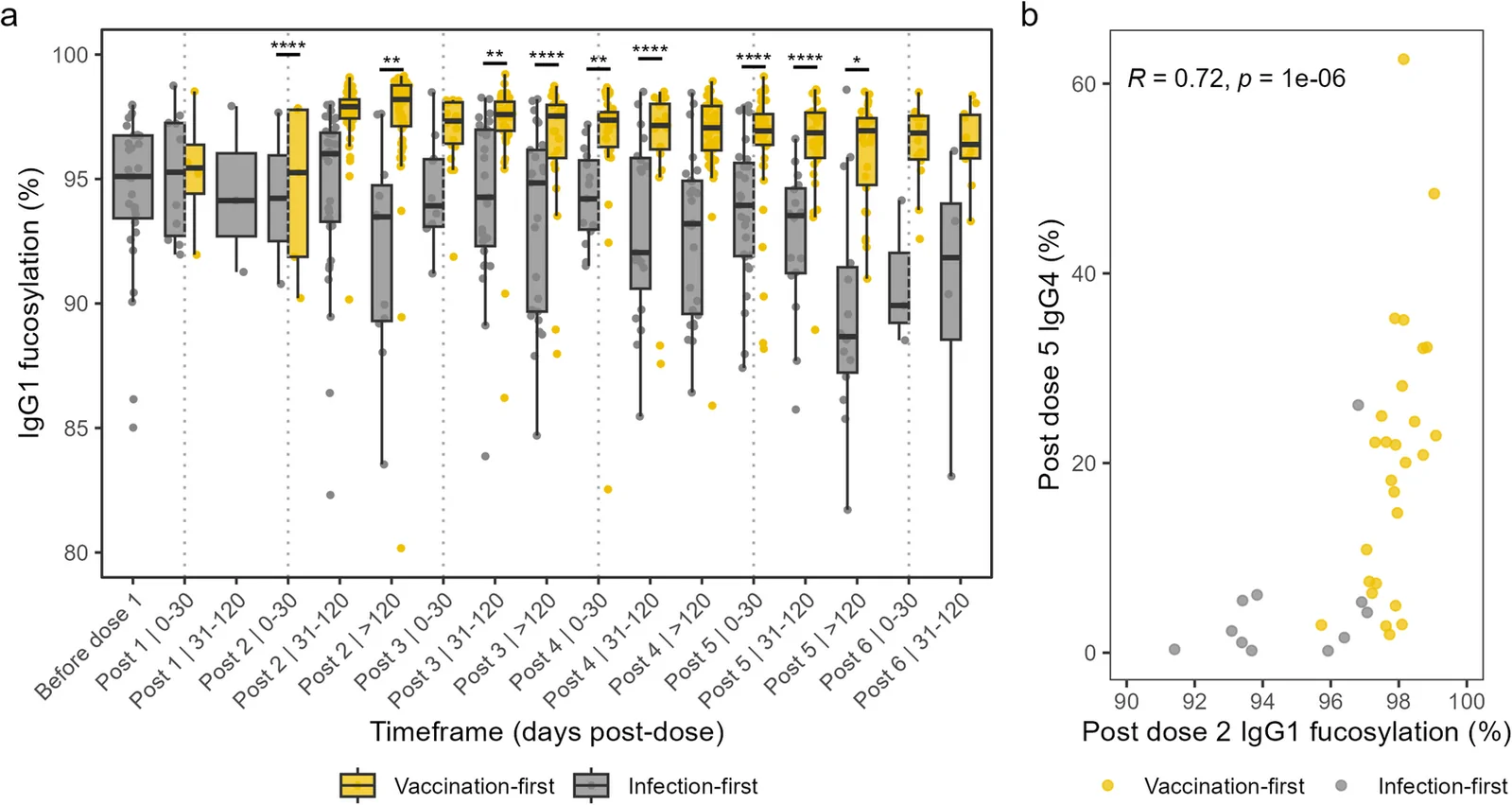
Anti-spike IgG1 fucosylation in vaccination-first and infection-first adults before the first and across 6 mRNA vaccine doses, and its correlation with spike-specific IgG4 responses. **(a)** Longitudinal spike-specific IgG1 fucosylation levels in vaccination-first (yellow) and infection-first (grey) adults across six mRNA vaccine doses. Between-group comparisons at each timepoint were performed using two-tailed Wilcoxon signed-rank tests. Non-significant p-values are not shown. Kruskal–Wallis test p-values were 0.04 for the naïve and 3 × 10^–8^ for the hybrid group. Vertical dotted lines denote the first timepoint after each vaccine dose. **(b)** Spearman correlation between early (post dose 2) IgG1 fucosylation levels and late (post 5) IgG4 proportions.

Building on our previous work demonstrating that infection prior to vaccination dictates Fc glycosylation changes following mRNA vaccination^34^, we investigated the long-term dynamics of anti-spike IgG1 Fc fucosylation in relation to prior SARS-CoV-2 infection. Extending this analysis over a multi-year timeframe and across six mRNA doses, we confirmed that the temporal pattern of IgG1 fucosylation is shaped by prior infection history: the vaccination-first group developed and sustained higher IgG1 fucosylation levels compared to the infection-first group, across the six mRNA doses (Fig. 4a).

Based on the observation that an IgG4 class switch and high IgG1 fucosylation was observed primarily in vaccination-first individuals (Fig. 4a), we assessed whether early IgG1 fucosylation levels correlated with the extent of IgG4 class switching. IgG1 fucosylation levels 31–120 days after the second vaccine dose were positively correlated with subsequent IgG4 proportions 31–120 days after the fifth dose (Fig. 4b). However, fucosylation levels alone, or after correction for IgG4 proportions, did not associate with breakthrough infection risk (Fig. S4).

### Booster vaccination triggers short-lived anti-spike galactosylation increases across IgG subclasses

IgG galactosylation levels were comparable across vaccination-first and infection-first individuals, with only minor differences observed for IgG2/3 and IgG4 at late timepoints following the fifth vaccine dose (Fig. S5). Of note, each booster dose induced a substantial but transient increase in galactosylation across all IgG subclasses, returning to the preceding levels by the later time points (>120 days) following each dose (Fig. S6). This is in line with previous observations after two to three mRNA doses^33^. When comparing IgG galactosylation levels between subclasses, we found that IgG2/3 and IgG4 consistently exhibited lower galactosylation than IgG1 (Fig. S7).

### Children display similar IgG4 class switch and Fc glycosylation patterns as adults

To assess whether the mRNA vaccination-induced IgG subclass and Fc glycosylation dynamics observed in adults arise also in pediatric populations, we analyzed samples from the exclusively vaccination-first MARVELS pediatric cohort who received two to three mRNA vaccine doses over the course of one year (Table 1, Fig. S8).

**Fig. 5:**
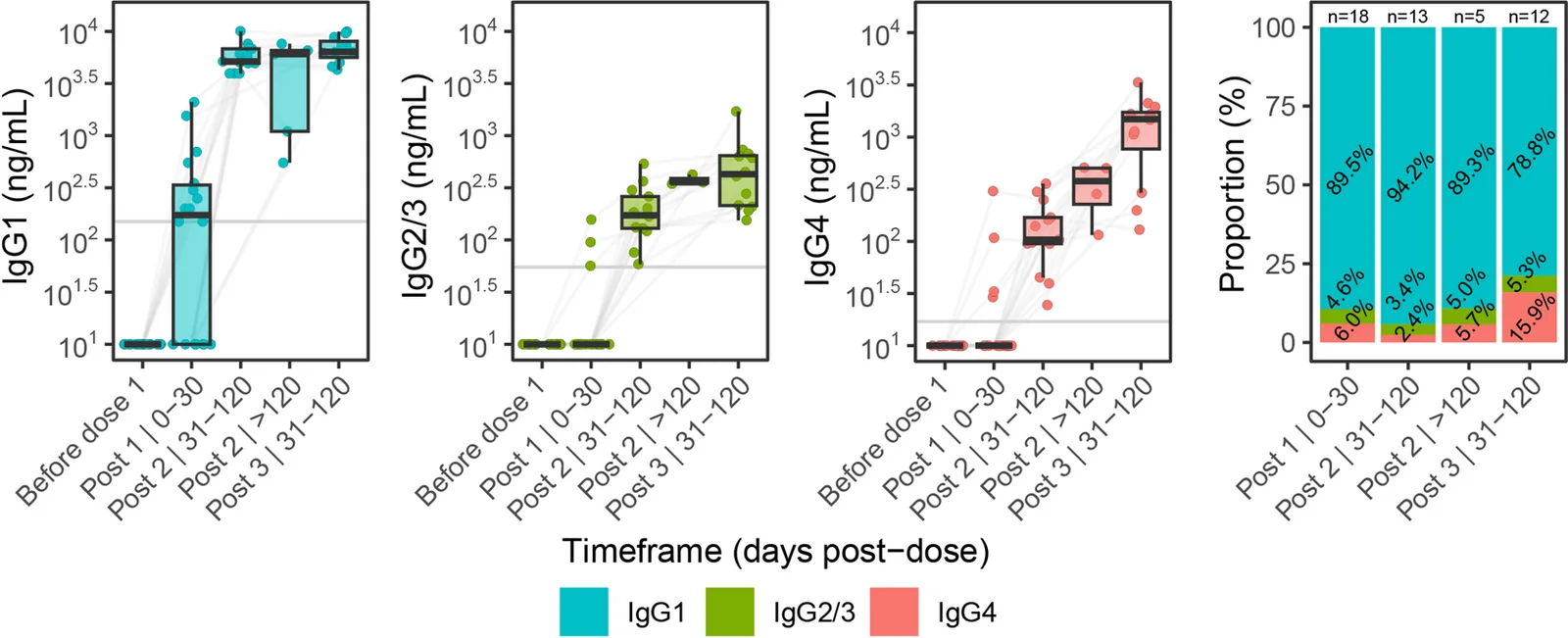
Subclass-specific IgG responses before and across 3 doses of mRNA vaccination in vaccination-first children. **(a–c)** Absolute concentrations of IgG subclasses following each vaccine dose, stratified by post-vaccination sampling timeframes: early (0–30 days), intermediate (31–120 days), and late (>120 days). Lower limit of quantification (LLoQ) indicated with horizontal lines for IgG1: 150 ng/mL; IgG2/3: 55 ng/mL; IgG4: 17 ng/mL. All post-vaccination timepoints were significantly higher relative to timepoints before vaccine 1, following correction for multiple comparisons using the Benjamini–Hochberg procedure (false discovery rate = 5%) of Wilcoxon signed-rank test derived p-values. **(d)** Proportional distribution of IgG subclasses per timeframe following each dose, expressed as percentages.

The build-up of IgG4, and corresponding subclass proportions, following the third vaccine dose, which is the time point at which IgG4 proportions plateaued in vaccination-first adults, were comparable in children and adults (15.9% and 18.1%, respectively (p > 0.8)) (Fig. 5a–d). In line with the adult cohort, IgG1 fucosylation increased after the second dose, while late galactosylation levels remained low across all subclasses relative to the early post-vaccination timepoint (Post 1| 0–30 days). IgG2/3 and IgG4 galactosylation remained substantially lower than IgG1 galactosylation (Fig. S9).

## Discussion

Building on previous reports describing an increased proportion of the IgG4 subclass following initial mRNA booster doses, we now show that this IgG4 class switch is sustained across at least six mRNA vaccine doses. Continuous infection monitoring in our adult cohort additionally revealed that an increased IgG4 proportion is associated with a higher breakthrough infection risk. Notably, the increase in IgG4 proportion was observed predominantly among individuals who were infection-naïve at the time of their first mRNA vaccine dose, emphasizing that immune priming by prior infection could influence subsequent vaccine-induced antibody responses. In addition, we show that IgG4 class switching correlates with elevated levels of IgG1 Fc fucosylation, a well-established anti-inflammatory Fc modification, early after primary mRNA vaccination, suggesting coordinated immune programming toward a potentially more tolerogenic antibody profile within the same individuals. Furthermore, we observed similar antibody structural features in a small pediatric cohort from a distinct geographical setting.

Several reports have demonstrated an IgG4 class switch upon repeated mRNA vaccination^14^. The mechanisms behind this class switch are not fully defined, but human studies have shown persistent germinal center reactions months following mRNA vaccination, implying sustained presentation of the spike glycoprotein^42,43^. This prolonged stimulus is a mechanistically plausible driver of a T_H_2-skewed, IL-10-rich milieu associated with IgG4 induction^31^, but current evidence remains correlative. IgG4-dominated responses have also been described for other high-frequency or high-antigen-load non-mRNA vaccine settings, including acellular pertussis, HIV and malaria vaccines^14^, as well as after infection with measles^44^ or Ebola^45^. Notably, the approved RTS,S/AS01 pediatric malaria vaccine induces IgG4 antibodies that can inhibit opsonophagocytic activity^46^. Analogously, sera enriched for anti-spike or anti-RBD IgG4 has been shown to attenuate Fc-mediated effector functions ^15,21,23,47,48^. These observations have contributed to the view that excessive IgG4 class switching may represent an unfavorable vaccine quality attribute, prompting questions about its long‑term evolution, clinical relevance and epidemiological impact. Notably, non-replicating adenoviral platforms^15,16,20^, live attenuated vaccines^28,49^, protein subunit formulations^18,19^ or heterologous vaccine regimens^15,16,19,50,51^ involving 3–4 doses have consistently been reported to induce minimal or no IgG4 class switching compared to repeated, closely spaced mRNA vaccinations, which may reflect lower cumulative antigen exposure.

Leveraging our immune profiling approach and infection surveillance of the longitudinal COMMUNITY HCW cohort, we were here able to compare longitudinal antibody trajectories between infection-first and vaccination-first adults, the latter previously shown to be most susceptible to IgG4 class switching^16,20,26^. Our results confirm and extend these earlier findings showing that pre-existing immunity from natural infection associates with lower magnitude of IgG4 class switching in response to repeated mRNA vaccination. Notably, IgG4 proportions remained elevated in participants who were SARS-CoV-2 naïve at the time of the first vaccine dose compared to individuals who had been infected prior to the first vaccine dose over the whole five-year follow up and across all six vaccine doses. Moreover, breakthrough infection or dosing schedule had negligible effect on IgG4 proportions, emphasizing that immune priming by infection prior to first vaccine dose may shape subsequent long-term vaccine-induced antibody responses. While the mechanisms by which immune priming by infection could attenuate IgG4 class switching upon subsequent mRNA vaccination remain to be elucidated, infection-induced T_H_1-skewed immune imprinting, favoring pro-inflammatory IgG subclasses while limiting an IgG4 class switch^14^, may contribute to this response.

Pérez et al. recently demonstrated that high IgG2 and IgG4 proportions were associated with an increased risk of breakthrough infection^29^, which is in alignment with our findings. However, unlike the study of Pérez et al.^29^, our correlates-to-protection analysis was restricted to vaccination‑first individuals without SARS‑CoV‑2 infection prior to the first vaccine dose since this was the only group in which we observed a significant IgG4 class switch. It is possible that combining vaccination‑first and infection‑first participants—as done by Pérez et al.^29^—may have diluted the IgG4 proportions and underestimated their association with breakthrough infection susceptibility. However, our findings remain suggestive and require validation in larger cohorts.

Correlates of protection against infection are also likely to differ with prior SARS-CoV-2 infection, as infection-induced mucosal IgA is expected to provide a substantial first line of defense in the upper respiratory tract^36–38^, which we recently showed overshadowed the contribution of systemic IgG^36^. Furthermore, the temporal sequence of infection and vaccination appears to influence mucosal IgA responses, with higher levels among participants with infection prior to systemic vaccination as compared to those with breakthrough infection as the first viral encounter^52^. Consequently, the association between a systemic IgG4 class switch and susceptibility to infection may be pronounced in vaccination-first individuals. Nevertheless, IgG4 has also been detected in both saliva and tears after mRNA vaccinations^23,53^, raising the possibility that increased IgG4 proportions may also influence protection at mucosal sites.

A related phenomenon has been described for pertussis, where repeated administration of acellular pertussis vaccines is associated with a shift towards high antigen‑specific IgG4 proportions that coincides with reduced vaccine effectiveness compared with whole‑cell or heterologous priming^54^, although a direct causal role of IgG4 in this context has not been demonstrated. The parallel nonetheless highlights that repeated exposure to a limited set of antigens reproducibly associates with IgG4 class switching across distinct vaccine platforms and pathogens.

It is important to emphasize that mRNA vaccination remains protective against severe COVID-19, and that the breakthrough infections in our cohort were asymptomatic or mildly symptomatic. While our data and those of Pérez et al.^29^ suggest a reproducible relationship between repeated mRNA vaccination, IgG4 class switching, and breakthrough infection risk, the functional relevance of this IgG4 shift cannot be deduced from the presented data in the absence of direct functional evidence. Furthermore, a potentially non-inflammatory IgG4 response may be compensated by Fab-dependent functions or T cell responses, while also potentially mitigating immunopathology. Nonetheless, the widespread use of repeated mRNA booster doses will merit from further longitudinal and mechanistic studies integrating systemic and mucosal antibody profiles, Fc‑effector functions, and clinical outcomes.

Given the extensive evidence that the monosaccharide composition of the conserved N‑glycan on the IgG heavy chain modulates both complement and FcγR‑mediated effector functions^9^, we extended our IgG subclass analyses to include longitudinal profiling of IgG1 Fc fucosylation and galactosylation.

Low-fucosylated (afucosylated) IgG1 responses are typically mounted against membrane-embedded antigens and have been associated with immunopathology in severe dengue virus infection and alloimmune responses^55^. Transient afucosylation of spike-specific IgG1 has been observed following severe SARS-CoV-2 infection, enhancing FcγRIII engagement and amplifying inflammatory cytokine production, inflammatory cell infiltration, antibody-dependent cellular cytotoxicity, and CD16⁺ myeloid cell activation^11,12,56–60^. Similar transient afucosylated responses occur shortly after mRNA vaccination in infection-naïve individuals, but not in those previously infected^33^. We therefore hypothesized that IgG1 Fc afucosylation might counterbalance the potentially anti-inflammatory IgG4 class switch observed in vaccination-first individuals.

Interestingly, and contrary to our expectations, long-term IgG1 Fc fucosylation levels remained increased in vaccination-first individuals as compared to infection-first individuals across at least six mRNA vaccine doses.

Building on this, we identify a correlative link between IgG1 Fc fucosylation and IgG4 subclass switching. High IgG1 Fc fucosylation after the second vaccine dose appears to serve as a surrogate marker of a subsequent IgG4 class switch. It is conceivable that elevated IgG1 Fc fucosylation coincides with regulatory humoral adaptations observed during repeated antigen exposure, including IgG4 class switching. In this context, increased fucosylation may contribute to reduced FcγRIII engagement and thereby subtly reinforce a shift toward lower Fc-mediated effector activity. However, although recent work has proposed high anti-dengue virus IgG1 Fc fucosylation as a correlate of vaccine‑induced protection against dengue breakthrough infection^61^, no association was observed between IgG1 fucosylation levels (adjusted for IgG4 proportions) and breakthrough infection risk in our cohort, rendering the observed minor differences most likely functionally irrelevant^32^.

Afucosylated IgG1 has been linked to improved control of HIV‑1 and protective immunity against malaria, underscoring its context‑dependent functional consequences^55,62^. Larger studies incorporating IgG subclass distribution and levels are therefore needed to clarify the potential role of IgG1 Fc fucosylation levels following repeatedly administered IgG4-inducing vaccines.

IgG1 Fc galactosylation enhances classical complement activation by promoting IgG hexamerization and C1q binding^63^. Counterintuitively, severe SARS-CoV-2 cases, along with a wide range of infectious^64^ and inflammatory diseases^65^, are characterized by markedly but transiently low antigen-specific IgG1 galactosylation levels^66^. This indicates that the complement overactivation observed in severe COVID-19 is unlikely to be driven by IgG galactosylation and may instead proceed predominantly through the alternative pathway^67^.

In sharp contrast to severe SARS-CoV-2 infections, transient increases in anti-spike IgG1 Fc galactosylation, along with the ability to activate the classical complement pathway through improved C1q binding, has been demonstrated following SARS-CoV-2 mRNA vaccination^19,33,34^, which is consistent with observations made upon influenza and tetanus vaccination^68^. We extend these observations by showing transient but substantial increases in Fc galactosylation across all IgG subclasses following each booster dose. Although a substantial IgG4 response could theoretically counteract IgG1/3 mediated complement activation on top of a potentially dampened ADCC, IgG4 is itself capable of inducing low-level complement activation under conditions with high antigen density and high galactosylation^69^. However, our findings display consistently lower IgG4 galactosylation levels compared to IgG1, rendering it unlikely that IgG4 galactosylation could rescue a potential IgG4-related dampening of immune effector functions.

Comparative analyses between adults and children is essential, particularly in the context of mRNA vaccine platforms^70,71^, which have emerged as a leading modality for pediatric vaccine development also against respiratory syncytial virus, influenza, human metapneumovirus, Epstein-Barr virus and cytomegalovirus^72^. The comparison of vaccination-first adult and pediatric populations in this study shows similar subclass and Fc glycosylation dynamics across up to three mRNA vaccine doses, including IgG4 emergence and long-term Fc glycosylation stability.

A major strength of this study is the comprehensive longitudinal documentation of both SARS-CoV-2 vaccinations and infections in an adult HCW cohort followed for five years. This extensive follow-up enabled detailed characterization of antibody structural features across six mRNA vaccine doses, while accounting for prior and intercurrent infections. Notably, samples were available before and between all six doses for all included participants, resulting in a uniquely well-resolved dataset. While our data demonstrate an IgG4 class switch consistent with previous reports^16,20^, and we did not detect an association between dose interval and IgG4 proportions, the modest variation in the interval between the first and second doses across groups represents a potential confounding factor that may have influenced group-level comparisons. Residual confounding by pandemic timing, circulating variants, vaccination intervals, and exposure history cannot be excluded when comparing infection-first and vaccination-first individuals. Furthermore, some degree of outcome misclassification cannot be excluded, but given the uniform surveillance framework and cohort characteristics, such misclassification is likely to be non-differential between groups. Despite the relatively large overall cohort, the subgroup of individuals without infection prior to first vaccination and until shortly after the third dose was limited in size and female only, which limited the statistical power to assess associations between IgG4 proportions and breakthrough infection risk, rendering these findings associative and non-generalizable. Nevertheless, the observed association with increased breakthrough infection risk is aligned with a prior report linking elevated IgG4 responses to an increased susceptibility to infection^29^. Participants received Omicron variant-matched boosters from the fifth vaccine dose onwards, whereas our affinity-capturing approach employed the ancestral spike protein. However, following monovalent ancestral priming and boosting, updated vaccine formulations have been reported to predominantly recall ancestral-type responses and/or induce spike-specific antibodies that cross-react with the ancestral spike^73^, unless individuals receive repeated boosting with variant-matched vaccines^74^. Thus, despite the use of ancestral spike for antibody capture, our approach likely captured a broad repertoire of spike-specific antibodies, including at later vaccination timepoints. Our methodological approach relied on a bottom-up glycoproteomics workflow that did not allow to distinguish between IgG2 and IgG3, and thus the exact balance between non-cytophilic and cytophilic subclasses. In addition, our analyses did not include long-term follow-up of individuals receiving heterologous booster regimens or those receiving only the primer mRNA vaccination series, although one study reports delayed induction of IgG4 responses in children after the second vaccination^75^. Future studies incorporating such comparator groups, more sex-balanced cohorts, and comprehensive mechanistic investigation will be important to fully assess the durability, potential functional relevance, and generalizability of mRNA-induced IgG4 responses. In the pediatric cohort, only vaccination-first children were included, precluding assessment of how prior infection may influence IgG4 class switching and antibody glycosylation patterns in children. Furthermore, the relatively small sample size limited our ability to evaluate associations between IgG4 proportions and breakthrough infection risk in this age group.

## Conclusions

mRNA vaccines have saved millions of lives worldwide and represent a highly promising vaccination platform due to their effectiveness, safety, scalability, and adaptability, with applications extending far beyond SARS-CoV-2^76^. As such, continued immunological monitoring is essential to support long-term optimization across populations and vaccine regimes. Here, we identify sustained IgG4 class switching following repeated mRNA vaccination in both adults and children, primarily in individuals without prior SARS-CoV-2 infection. This structural antibody adaptation was associated with an increased breakthrough infection susceptibility in a limited set of individuals. A more detailed understanding of how prior infection shapes vaccine-induced immunity, and antibody structures such as IgG subclass and glycosylation relate to protection may inform the rational refinement of vaccination regimens, including dosing strategies and heterologous approaches.

## Methods

### Cohort 1 (adult cohort)

The Covid-19 Immunity (COMMUNITY) HCW cohort (NCT06784739) comprises 2149 HCW, aged 30–69, who have been followed every four months since April 2020. Blood samples were collected through venipuncture, and clinical information was collected through a smart phone-based application. All HCW were offered vaccination starting in January 2021. SARS-CoV-2 infection prior to or following vaccination was confirmed by seroconversion and/or by qPCR, but some degree of outcome misclassification, as inherent to any observational study, cannot be excluded. Data regarding which vaccine and date of vaccination was obtained through the Swedish vaccination register (VAL Vaccinera) and data regarding PCR-confirmed SARS-CoV-2 infection was obtained through the national communicable disease surveillance register SmiNet (Public Health Agency of Sweden). Antibody responses were determined in four-month intervals since enrolment or more frequently within PCR screening substudies^36–38,77–79^. In this study, we selected 104 immunocompetent HCWs with at least 5 administered mRNA vaccines doses (30 or 10 µg for BNT162b and mRNA-1273 including variant-matched boosters, respectively). Samples were selected as follows: i) one pre-vaccination sample, ii) one sample within day 1–6 post each dose or iii) one sample post each dose (from day 7 and onward, the first one chosen), and iv) the last available sample post each dose, resulting in a total of 831 samples. All study participants provided written informed consent, the study protocol was approved by the Swedish Ethical Review Authority (dnr 2020–01653), and all research was performed in accordance with relevant guidelines/regulations and with adherence to the Declaration of Helsinki. See Tables 1 and S2 for detailed cohort characteristics and Fig. S1 for the sampling time distribution.

### Cohort 2 (pediatric cohort)

The MARkers of Vaccine Efficacy and Longevity in SARS-CoV-2 (MARVELS) cohort comprised 110 healthy Singaporean children, aged 5–12, recruited between 20 December 2021 and 8 March 2022 and followed up for 1 year. We selected 18 children who received two to three doses of 10 µg BNT162b2 and had neither clinical history nor serological or T cell evidence of previous SARS-CoV-2 infection for this study^80^. The vaccination period coincided with the emergence of the Omicron variant in Singapore, at a time when most of the adult population had already been fully vaccinated. Samples were selected based on availability. This cohort provided a unique opportunity to compare IgG class switch and Fc glycosylation in children to the same parameters found in adults. The study protocol was approved by the National Healthcare Group Domain Specific Review Board (DSRB) (2021/00945 and 2021/00984), and all research was performed in accordance with relevant guidelines/regulations and with adherence to the Declaration of Helsinki. Furthermore, informed consent was obtained from a parent or legal guardian of all participants. See Table 1 for detailed cohort characteristics and Fig. S2 for the sampling time distribution.

### Subclass-specific IgG quantification and Fc glycosylation analysis

Subclass-specific IgG were quantified using a liquid chromatography-mass spectrometry (LC–MS) approach relying on stable isotope-labeled, subclass-specific internal IgG standards mixed with anti-spike antibodies after purification with an immunosorbent assay. This quantitation method correlates well with established enzyme-linked immunosorbent assays^35^. Briefly, spike-specific antibodies were captured by the GlycoLISA approach using ancestral spike protein-coated plates^81^. 96-well Maxisorp NUNC-Immuno plates (Thermo Fisher) were separately coated with a concentration of 1 μg/mL antigens at 4 °C overnight. After washing with 0.05% Tween-20 in PBS, tenfold diluted serum was first added to the plate and incubated for 1 h while shaking at room temperature. Serum-incubated plates were washed three times with PBS with 0.05% Tween, twice with PBS and twice with 50 mM ammonium bicarbonate. Spike-bound antibodies were eluted from the plate using 100 mM formic acid and then spiked with the SILuMAB IgG1 and IgG4 heavy isotope-labeled antibody standards (Sigma-Aldrich)^35^. Heavy isotope-labeled IgG2 and IgG3 subclasses were not commercially available at the time of conducting the study. The eluates were dried by vacuum centrifugation. Tryptic cleavage of IgG and subsequent LC–MS (glyco-)peptide measurements were carried out as described previously^81^. Briefly, (glyco-)peptides were separated on a NanoEase M/Z peptide BEH C18, 1.7 μm particles 130 Å pores, 75 μm × 100 mm (Waters) at a flow rate of 600 nL/min using a binary gradient of 0.02% aqueous TFA and 95% acetonitrile. The nanoLC-ESI–MS setup combined an Ultimate 3000 LC (Thermo Fisher) with an Impact time-of-flight MS (Bruker Daltonics) via a CaptiveSpray source using acetonitrile-enriched nitrogen nebulizer gas (3 L/min at 180 °C).

MS signals of (glyco-)peptides were quantified by integrating MS peak areas after generating sum spectra of the retention time ranges of interest. Raw LC–MS data files were converted to mzXML files using ProteoWizard MSConvert (version 3.0.23037). The mzXML files were processed in LaCyTools version 2.1.0 (build 20,230,525), using calibration at a signal-to-noise (S/N) cut-off of 9. GlycoDash (version 1.6.5; https://github.com/Center-for-Proteomics-and-Metabolomics/glycodash) was used to perform spectral and analyte curation, total area normalization and calculation of glycosylation traits (Table S3). Combined IgG2/3 quantities could be reliably estimated by dividing the summed IgG2/3 proteotypic glycopeptide abundances by the summed IgG4- or IgG1-derived heavy isotope-labeled proteotypic glycopeptide abundances. IgG2 and IgG3 subclasses share the same tryptic glycopeptide backbone and thus could not be distinguished using the employed LC–MS method. The assumed peptide backbones for IgG1, IgG2/3 and IgG4 were EEQYNSTYR, EEQFNSTFR, EEQFNSTYR, respectively.

Vaccination-first individuals were used as controls during MS spectra curation, where we used the 90th percentile as a lower limit of quantification (IgG1: 150 ng/mL; IgG2/3: 55 ng/mL; IgG4: 17 ng/mL). Datapoints under the LLoQ cut-off and/or participants seronegative based on spike-targeting ELISA prior to or within 7 days after vaccine dose 1 were set to 10 for visualization and statistical purposes. Any other datapoints that fall below the LLoQ cut-off 7 days after vaccination were considered technical and excluded from analysis. An analyte was included in the final data analysis when it fulfilled three quality criteria (S/N > 9, isotopic pattern quality < 0.2, absolute mass error < 20 ppm) in at least 80% of adult and/or child sample spectra. For a list of quantified glycopeptide analytes see Table S4.

### Statistical analysis

Continuous variables were displayed on boxplots with whiskers representing the first and third quartiles. Wilcoxon-signed rank test or post-hoc Dunn’s test results (following Kruskal–Wallis test) were used for group comparisons following correction for multiple testing using the Benjamini–Hochberg procedure at a FDR of 5%. Only significant associations were shown, with significance levels as follows: *p < 0.05, **p < 0.01, ***p < 0.001, respectively. Datapoints under the LLoQ cut-off and/or for participants seronegative prior to the first vaccination based on serology were set to 10 for visualization and statistical purposes (Fig. 1). Hazard ratios were estimated using multivariable Cox proportional hazards models (Fig. 3). A Spearman’s rank correlation analysis was performed to assess the strength and direction of the association between IgG1 fucosylation levels and IgG4 levels. All statistical analysis and visualizations were performed in RStudio (version 2024.12.1+563) and R (version 4.5.0) using the packages: ‘Table1’, ‘tidyverse’, ‘ggprism’, ‘ggpubr’, ‘ggbeeswarm’, ‘rstatix’, ‘survival’, ‘survminer’, ‘broom’ and ‘scales’.

## Supplementary Information

Below is the link to the electronic supplementary material.

Supplementary Material 1.

Supplementary Material 2.

## Data Availability

Anonymized participant data will be made available to qualified researchers upon reasonable request to the corresponding authors subject to approval by the institutional review board and a data use agreement. Data will only be made available for academic, non-commercial purposes. Supporting materials such as the study protocol and statistical code are available upon request.
