# Supplementary material for: Vaccination-first immune priming shapes a sustained mRNA vaccine-induced IgG4 class switch that associates with SARS-CoV-2 breakthrough infection risk
Source: Sci Rep. 2026 Aug 12;16:26038. doi: 10.1038/s41598-026-64190-8 (PMC13490519; doi:10.1038/s41598-026-64190-8)
Supplement: Supplementary file 1 [file 41598_2026_64190_MOESM1_ESM.pdf]

SUPPLEMENTARY MATERIAL ASSOCIATED WITH THE WORK:

Vaccination-first immune priming shapes a sustained mRNA vaccine-induced IgG4 class switch that associates with SARS-CoV-2 breakthrough infection risk

Tamás Pongrácz[1], Ulrika Marking[1], Oscar Bladh[1], Katherina Aguilera[1], Matilda Berkell[1,2], Sebastian Havervall[1], Nina Greilert-Norin[1], Jan Nouta[3], Steinar Gijze[3], Anna Wasynczuk[3], Mikael Åberg[4,5], Gestur Vidarsson[6,7], Youjia Zhong[8,9], David Falck[3], Charlotte Thålin[1]

[1]Department of Clinical Sciences, Karolinska Institutet, Danderyd Hospital, Stockholm, Sweden

[2]National Bioinformatics Infrastructure Sweden, Stockholm, Sweden

[3]Center for Proteomics and Metabolomics, Leiden University Medical Center, Leiden, Netherlands

[4] Department of Medical Sciences, Clinical Chemistry, Uppsala University, Uppsala, Sweden

[5]Science for Life Laboratory, Uppsala University, Uppsala, Sweden

[6]Immunoglobulin Research laboratory, Sanquin Research, Amsterdam, Netherlands

[7]Department of Biomolecular Mass Spectrometry and Proteomics, Utrecht Institute for Pharmaceutical Sciences and Bijvoet Center for Biomolecular Research, Utrecht University, Utrecht, Netherlands

[8]Department of Paediatrics, Yong Loo Lin School of Medicine, National University of Singapore, Singapore, Singapore

[9]Khoo Teck Puat-National University Children's Medical Institute, National University Health System (NUHS), Singapore, Singapore

TABLE OF CONTENTS

Figure S1. Exploratory correlation analyses between IgG4 proportions and vaccine dose intervals within 30 days after doses 3, 4 and 5 in adults.

Figure S2. Influence of breakthrough infection on long-term IgG4 proportions, as assessed on paired samples between vaccine dose 3 and vaccine dose 5 in vaccination-first and infection-first adults.

Figure S3. Visual overview of the sampling times relative to each vaccine dose.

Figure S4. Cumulative incidence of breakthrough infections over a 200-day follow-up period in adults with mixed immune histories (both vaccination first and infection-first) after the third vaccine dose, stratified into groups with “high” and “low” fucosylation levels divided at 95%.

Figure S5. Subclass-specific anti-spike IgG galactosylation responses across six mRNA vaccine doses in adults.

Figure S6. Comparison of subclass-specific anti-spike IgG galactosylation responses upon the second dose of the primary vaccination regimen and subsequent booster doses in adults.

Figure S7. Comparison of anti-spike IgG galactosylation responses between subclasses upon the second dose of the primary vaccination regimen or a subsequent booster dose in adults.

Figure S8. Visual overview of the sampling times relative to each vaccine dose within the The MARkers of Vaccine Efficacy and Longevity in SARS-CoV-2 (MARVELS) childrens cohort.

Figure S9. Comparison of anti-spike IgG1 fucosylation and subclass-specific IgG galactosylation responses across 3 mRNA vaccine doses in children.

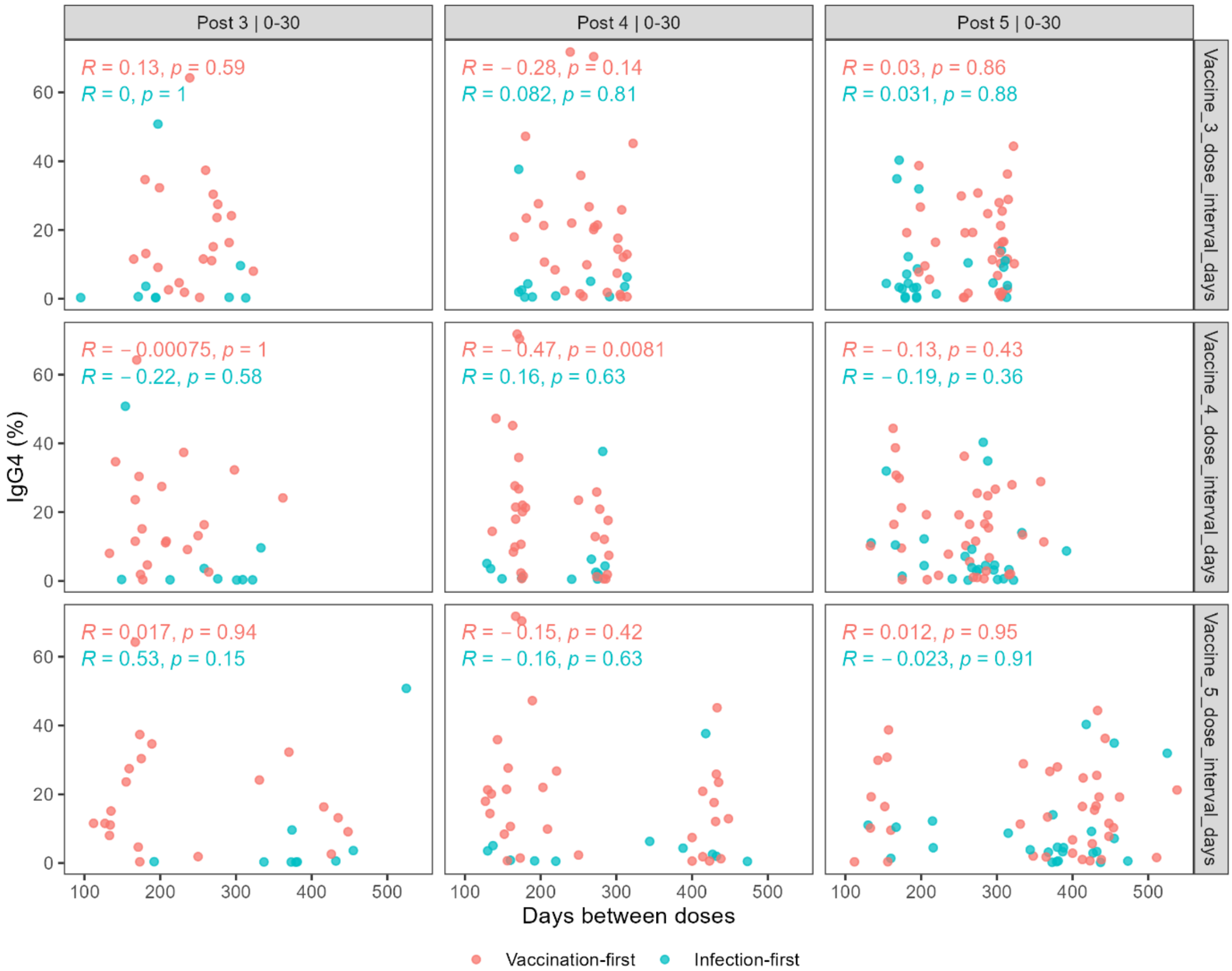

Figure S1. Exploratory correlation analyses between IgG4 proportions and vaccine dose intervals within 30 days after doses 3, 4 and 5 in adults. No consistent trend was found, indicating that the dose intervals did not influence IgG4 proportions in this study. The only significant negative correlation found was for the vaccination-first group following dose 4. We found the dose 1-2 interval lower in vaccination-first (median [IQR] = 21 [21-22]) than in infection-first individuals (median=40 [21-43]), but early timepoints were not available to perform a similar analysis. However, we found no correlation between dose day interval and IgG4 proportions in the timeframes 31-120 and >120 days after dose 2, when IgG4 proportions started to emerge. This indicates that the dose interval during the primary vaccination regimen also did not influence IgG4 proportions.

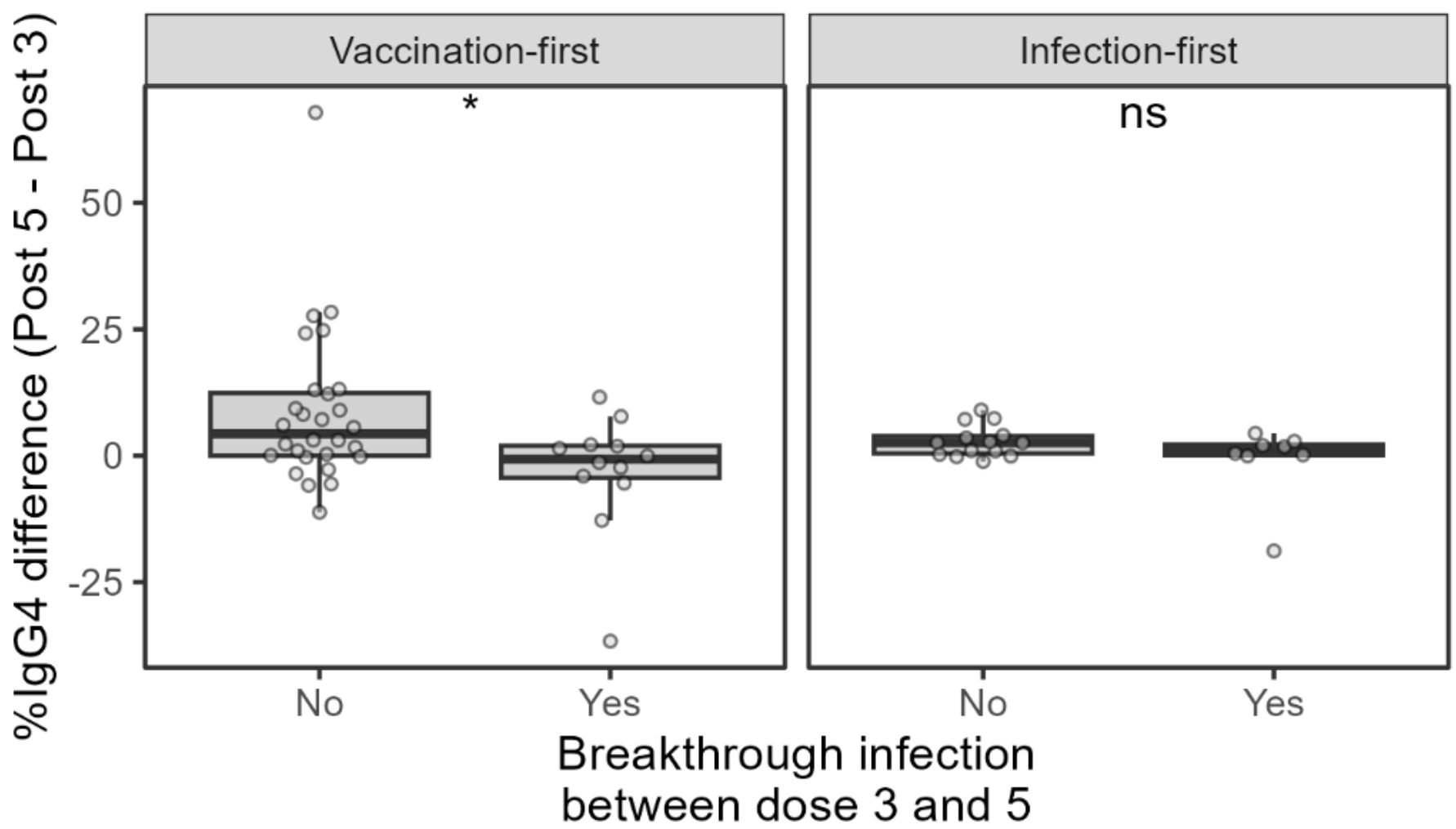

Figure S2. Influence of breakthrough infection on long-term IgG4 proportions, as assessed on paired samples between vaccine dose 3 and vaccine dose 5 in vaccination-first and infection-first adults. The y-axis shows the %IgG4 difference between dose 5 and dose 3. Significance levels: * $p < 0.05$; ns: not significant. A significant but in magnitude negligible effect was shown in vaccination-first individuals, indicating that breakthrough infections likely did not affect long-term IgG4 proportions.

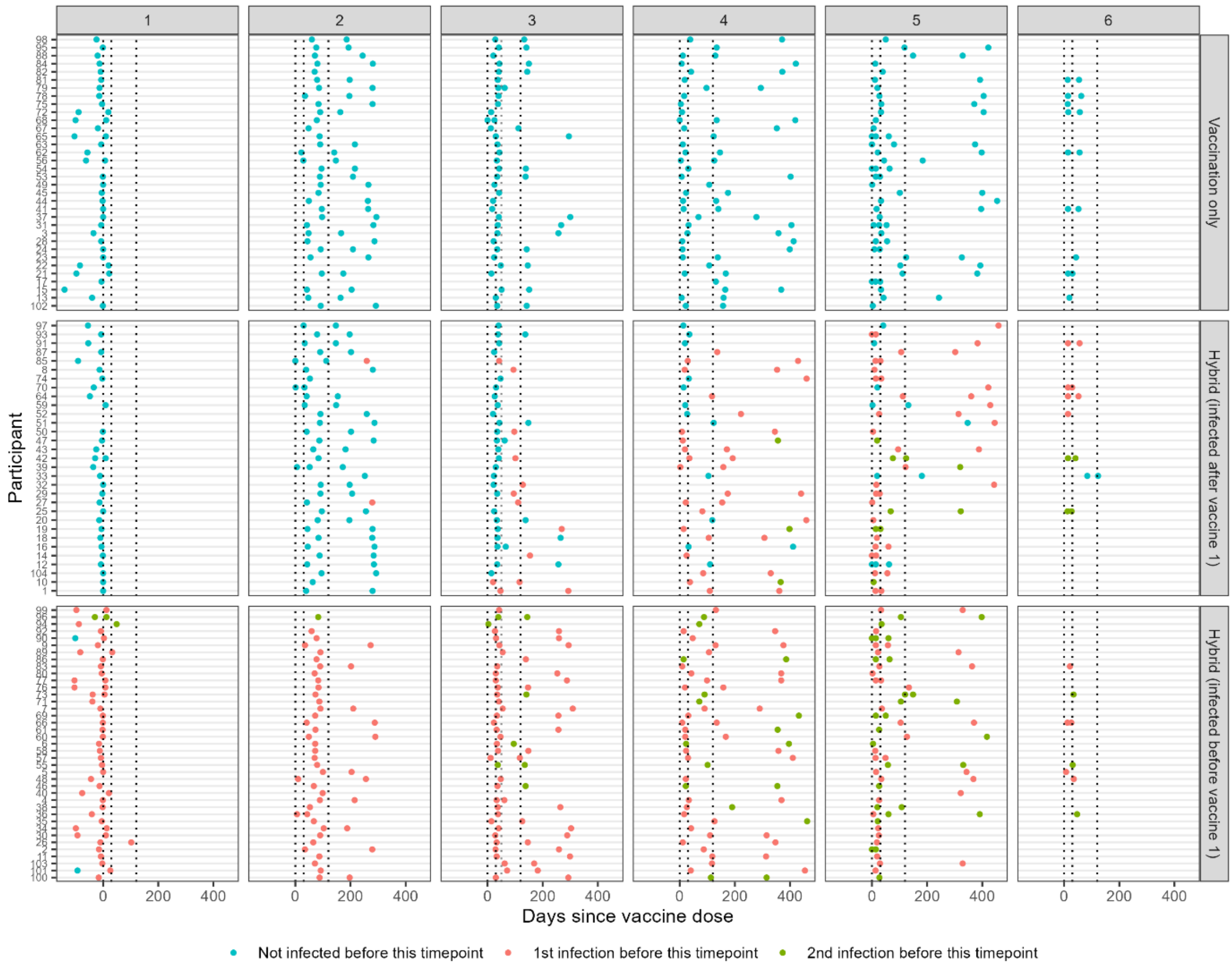

Figure S3. Visual overview of the sampling times relative to each vaccine dose. The HCWs were stratified into three groups depending on immune history: Vaccination only; Hybrid (infected after vaccine 1) and Hybrid (infected before vaccine 1). Colors indicate infection history up until the sampling (blue: no infection; red: one infection; green: two infections). Black dotted vertical lines indicate time of vaccination (day 0), 30 and 120 days, illustrating the three subgroups generated for **Figures 1, 2, 4** (0-30; 31-120; >120)

after each dose. Uninfected individuals left of the grey vertical dotted line crossing at 50 days were included in the survival analysis displayed in **Figure 3**. Two individuals who received a seventh vaccine were not included into analyses due to ineligibility for statistical comparisons.

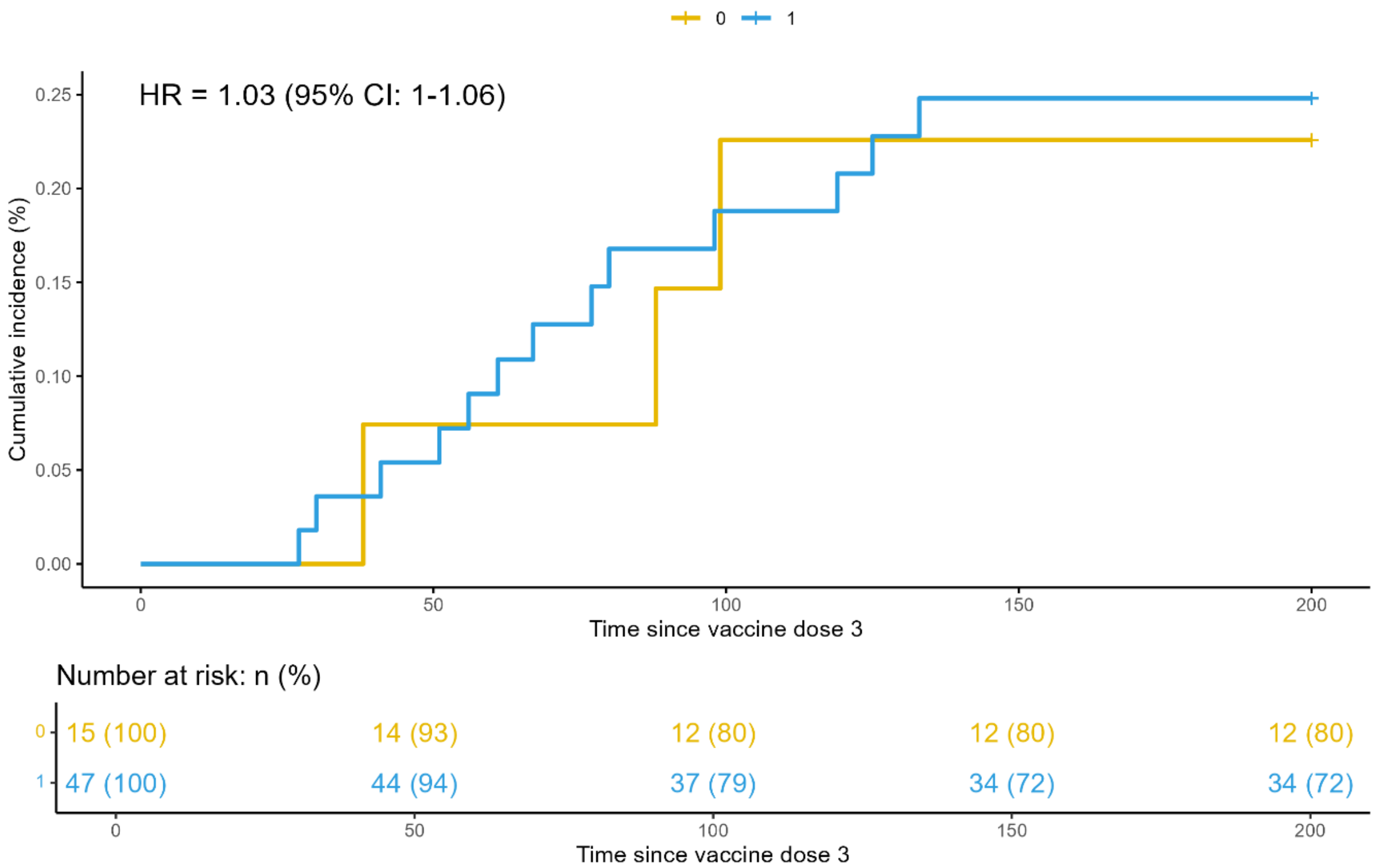

Figure S4. Cumulative incidence of breakthrough infections over a 200-day follow-up period in adults with mixed immune histories (both vaccination first and infection-first) after the third vaccine dose, stratified into groups with "high" and "low" fucosylation levels divided at 95%. High IgG1 fucosylation was considered as an exposure (1), whereas low fucosylation as non-exposure (0). The rationale behind this choice was the broadly described negative association between IgG1 Fc fucosylation and inflammatory potential. Hazard ratio was estimated using Cox proportional hazards models. IgG4 proportion has been included as a covariate into the model, but neither IgG1 fucosylation alone nor after correction to IgG4 proportions showed significant association with breakthrough infection risk.

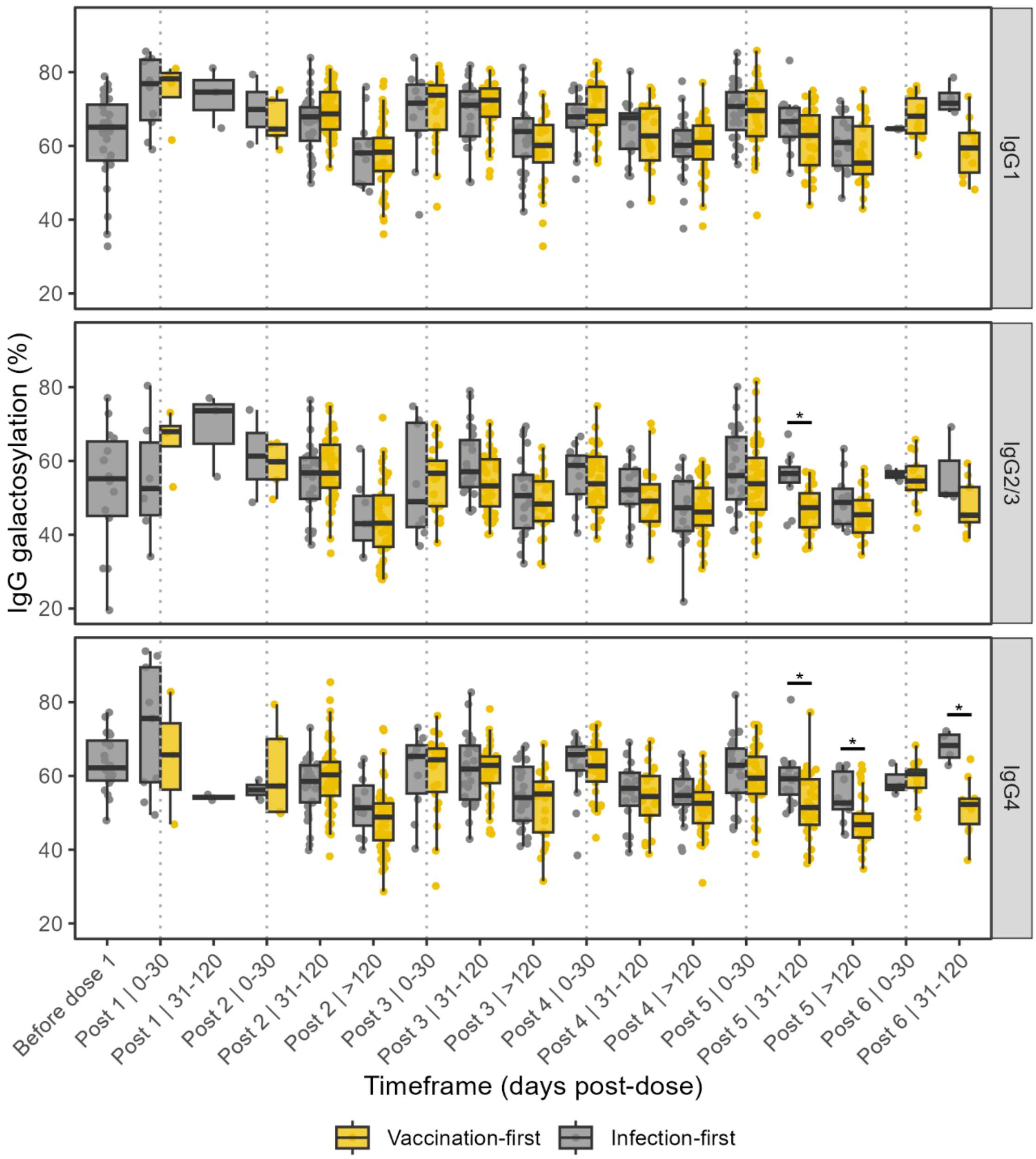

Figure S5. Subclass-specific anti-spike IgG galactosylation responses across six mRNA vaccine doses in adults. Vaccination-first and infection-first groups were compared at each timepoint using two-tailed Wilcoxon signed-rank tests. Dotted vertical lines indicate the first sampling timepoint after each vaccine dose. The analysis suggests no difference between groups over time. Significance levels: * p < 0.05. Only significant associations are shown.

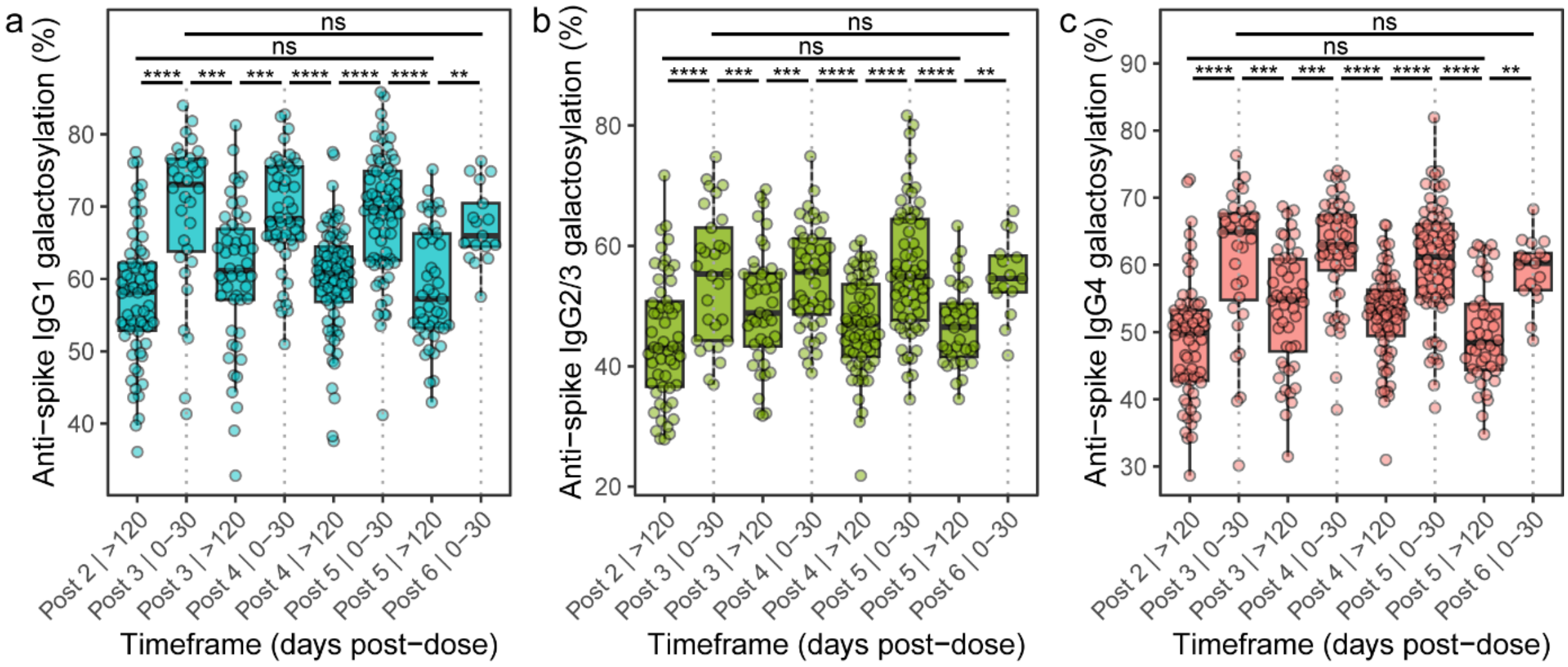

Figure S6. Comparison of subclass-specific anti-spike IgG galactosylation responses upon the second dose of the primary vaccination regimen and subsequent booster doses in adults. Infection-first and vaccination-first groups were assumed to be not different based on **Figure S5** and thus have been combined in this analysis. Selected groups pre- and post-vaccination were compared at each timepoint using two-tailed Wilcoxon signed-rank tests. Dotted vertical lines indicate the first sampling timepoint after each vaccine dose. This analysis shows a clear but transient effect of booster vaccination on IgG galactosylation across all subclasses. Significance levels: **, ***, **** p < 0.01, 0.001, 0.0001; ns: not significant.

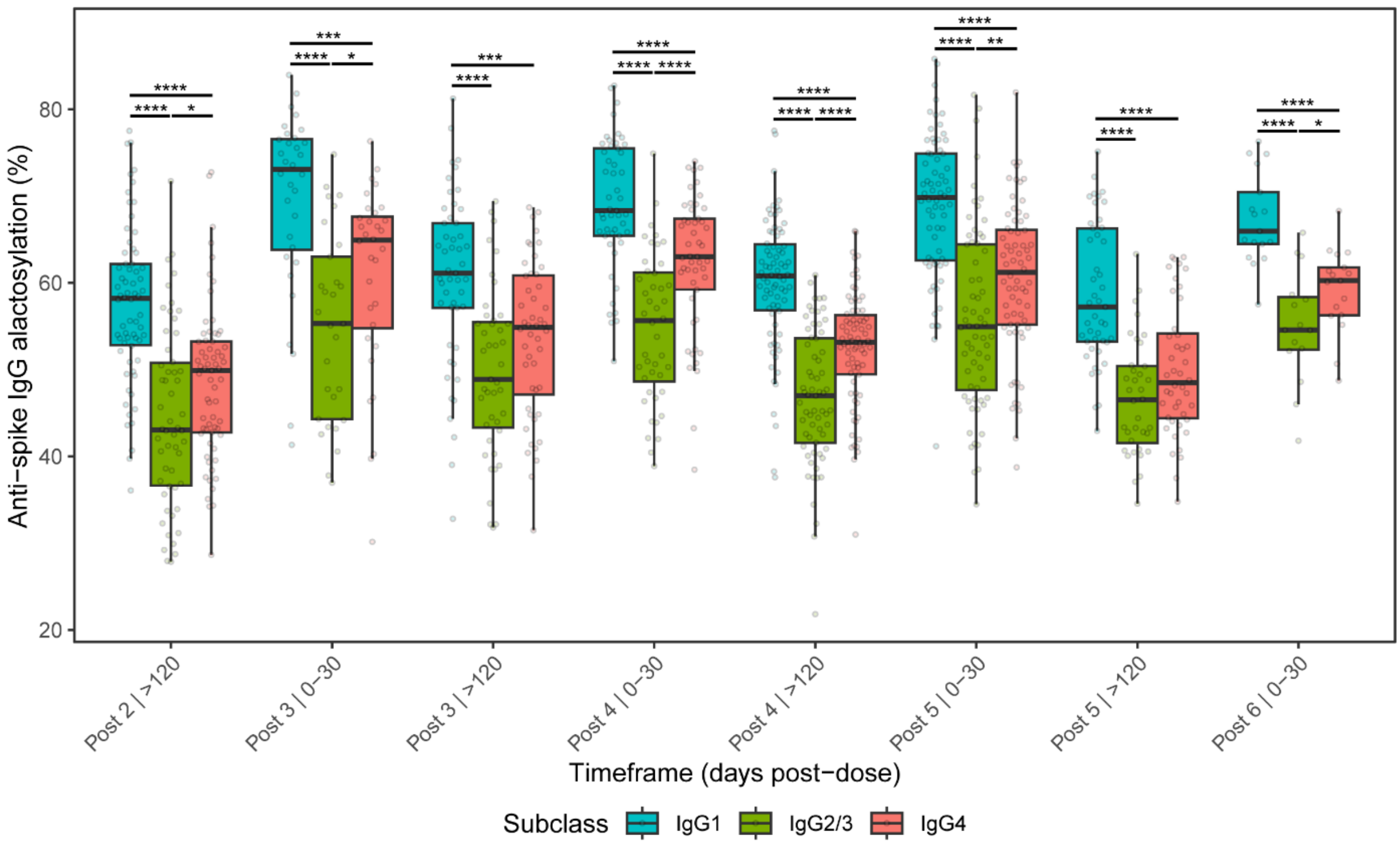

Figure S7. Comparison of anti-spike IgG galactosylation responses between subclasses upon the second dose of the primary vaccination regimen or a subsequent booster dose in adults. Infection-first and vaccination-first groups were assumed to be not different based on **Figure S5** and thus have been combined in this analysis. Selected groups pre- and post-vaccination were compared at each timepoint using two-tailed Wilcoxon signed-rank tests. Significance levels: *, ***, **** p < 0.05, 0.001, 0.0001. Only significant associations are shown. This analysis shows that IgG1 galactosylation consistently exceeds those of IgG2/3 and IgG4, respectively.

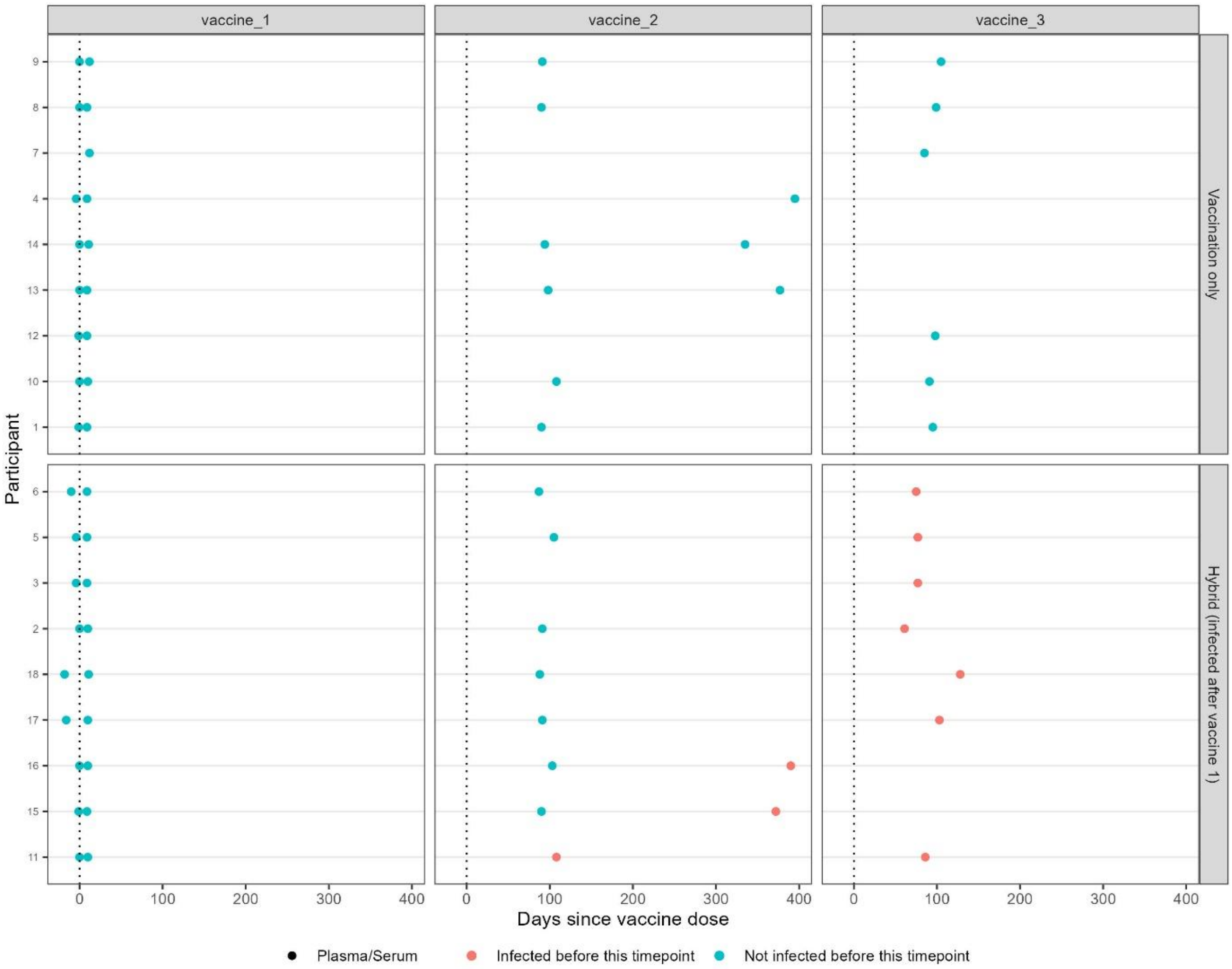

Figure S8. Visual overview of the sampling times relative to each vaccine dose within the The MARkers of Vaccine Efficacy and Longevity in SARS-CoV-2 (MARVELS) pediatric cohort.

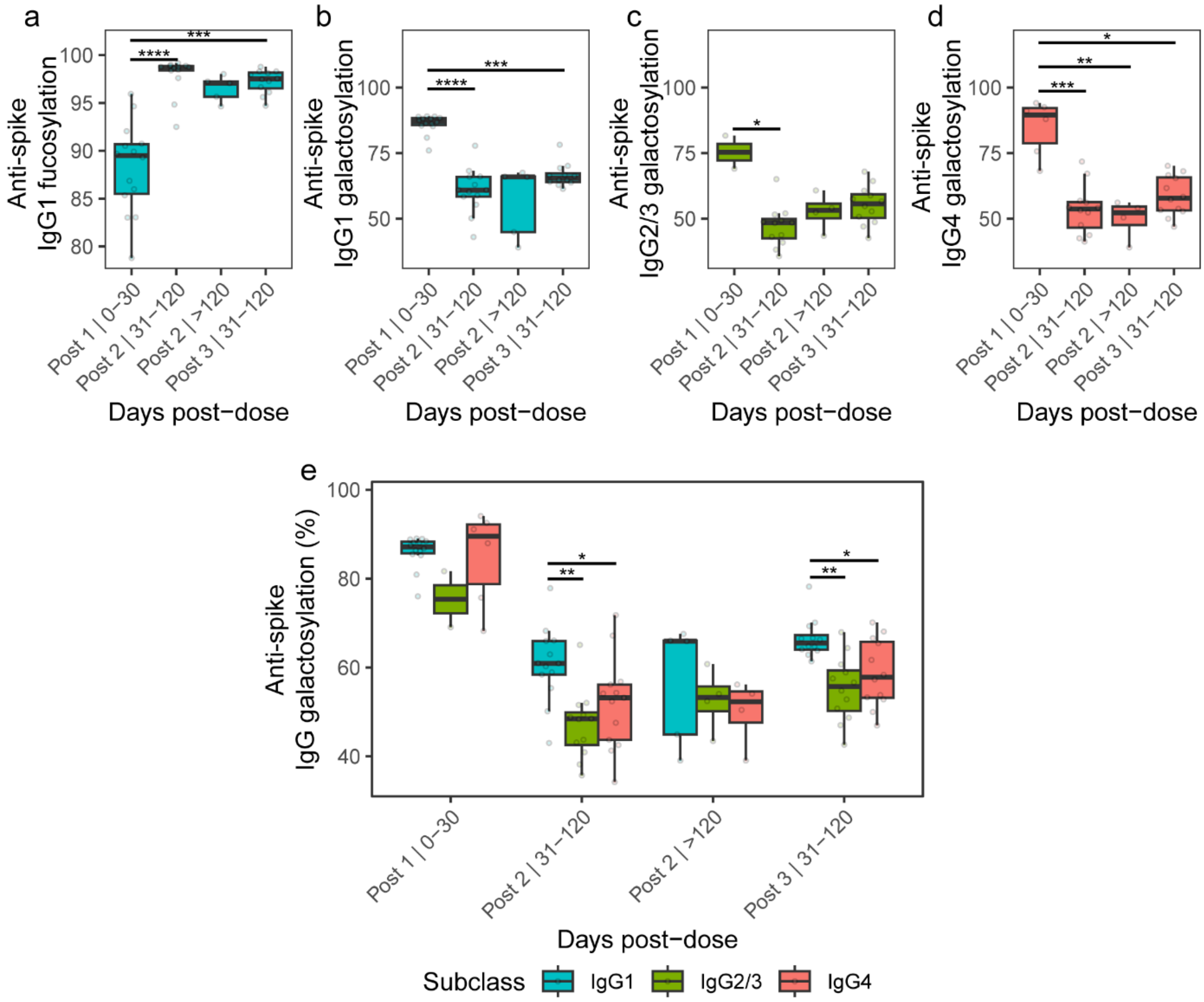

Figure S9. Comparison of anti-spike IgG1 fucosylation and subclass-specific IgG galactosylation responses across 3 mRNA vaccine doses in children. Selected groups were compared at each timepoint using two-tailed Wilcoxon signed-rank tests. Significance levels: *, **, ***, **** p < 0.05, 0.01, 0.001, 0.0001; ns: not significant. This analysis shows similar glycosylation patterns and that IgG1 galactosylation consistently exceeds those of IgG2/3 and IgG4 in children as in adults.
